# Population Genetics of the Moon Crab *Matuta victor* (Fabricius 1781) on Java Island, Indonesia

**DOI:** 10.21315/tlsr2026.37.1.9

**Published:** 2026-03-31

**Authors:** Nur Ikhlas Syuhada, Achmad Farajallah, Ali Mashar

**Affiliations:** 1 Department of Biology, Faculty of Mathematics and Natural Sciences, IPB University, Kampus IPB Dramaga, Bogor, 16680 West Java, Indonesia; 2 Department of Aquatic Resources Management, Faculty of Fisheries and Marine Sciences, IPB University, Kampus IPB Dramaga, Bogor, 16680 West Java, Indonesia; 3 Department of Biology, Faculty of Mathematics and Natural Sciences, Universitas Riau, Kampus Bina Widya, Pekanbaru, 28293 Riau, Indonesia

**Keywords:** *COI* Mitochondria, Genetic Diversity, Moon Crab, Population Genetic

## Abstract

Genetic diversity is crucial in assessing biodiversity and is increasingly being utilised in conservation and management. This study aimed to analyse the population genetics of *Matuta victor* on Java Island. A total of 91 specimens was collected from 10 localities: Ujung Kulon, Pangandaran, Carita, Tanjung Pasir, Subang, Bancar, Banyuates, Ambunten, Situbondo and Banyuwangi. We obtained 801 bp fragments of *COI* mitochondrial DNA defining 38 different haplotypes, namely, 12 shared and 26 unique haplotypes. H1 was the most frequent, existing in eight localities. The haplotype diversity (Hd) and nucleotide diversity (π) ranged from 0.667 to 1 and 0.003 to 0.022, respectively. The overall haplotype diversity was high (Hd = 0.921), indicating the presence of many haplotypes across populations, while the overall nucleotide diversity was low (π = 0.018), suggesting only small genetic differences at the nucleotide level. AMOVA analysis showed a low level of genetic differentiation among the 10 locations (FST = 0.266). Neutrality tests indicated the potential population expansion of *M. victor* in several locations in Java. These findings are expected to constitute a significant first step in designing targeted and sustainable conservation strategies for this species in its diverse coastal habitats in Indonesia.

**HIGHLIGHTS**

- A total of 38 *COI* haplotypes of *Matuta victor* were identified from 91 individuals collected across 10 coastal sites on Java Island, indicating high haplotype diversity (Hd = 0.921).
- Low nucleotide diversity (π = 0.018) and AMOVA results (FST = 0.266) revealed limited genetic differentiation among populations, suggesting extensive gene flow and genetic homogeneity across coastal areas of Java.
- Neutrality tests indicated potential population expansion in several locations, providing baseline genetic information for conservation and management of *M. victor* in Indonesia’s coastal ecosystems.

## INTRODUCTION

Java Island harbours a high diversity of brachyuran crabs across various coastal habitats (Irwansyah *et al*. 2021; Maryani *et al*. 2023), although a comprehensive species checklist for the island is still lacking. Within this rich diversity, the moon crab *Matuta victor* stands out because of its wide distribution, ecological role as a benthic predator (Galil & Mendelson 2013), keystone species (Hanim *et al*. 2021) and potential as a bioindicator (Wisespongpand *et al*. 2024). These characteristics make *M. victor* a suitable model species for understanding population genetics in coastal environments of Indonesia.

The moon crab, *Matuta victor*, is a member of the family Matutidae that inhabits sandy beaches (Ates *et al*. 2017). This species has a wide distribution, ranging across the Mediterranean Sea, Kasos Island, Gulf of Suez, Red Sea, Gulf of Oman, Arabian Sea, Comoro Islands, Madagascar, India, Andaman Sea, Malaysia, Indonesia, South China Sea, Japan, Australia, New Caledonia and New Hebrides (Galil & Mendelson 2013; Behera *et al*. 2018; Fortic *et al*. 2023).

Understanding a species historical and current population structure is crucial for studying evolutionary processes such as genetic drift, selection and adaptation. Molecular comparisons can reveal genetic separations between populations and complex phylogeographic structures within and among different populations (Yednock & Neigel 2011). Using genetic markers, for example, the *COI* gene, enables us to more accurately estimate the level of genetic exchange between populations based on empirical evidence (Hellberg 2009). Indonesia is flanked by the Indian and Pacific Oceans. Currents from the Western Pacific connect with the Indonesian Throughflow, which transports warm water through the Java Sea into the Indian Ocean (Lee *et al*. 2002; Schonau *et al*. 2015).

Genetic diversity is a critical component of biodiversity assessment and is increasingly used in conservation and management implementation (Goodall-Copestake *et al*. 2012). Despite its wide distribution and ecological role, little is known about the population genetics of *M. victor* in Indonesia. Previous studies have mostly reported occurrence records, but genetic data remain scarce. This study presents the first report on the population genetics of *M. victor* and addresses this gap by analysing its genetic diversity and population structure in Java Island. By understanding the distribution patterns and genetic variation of *M. victor*, valuable insights can be provided for more effective conservation and management efforts. Thus, this study provides important baseline information on the genetic diversity and structure of *M. victor* populations in Java.

## MATERIALS AND METHODS

### Sample Collection

Between 2016 and 2023, a total of 91 specimens were collected from 10 locations on Java Island (Table 1). Samples were collected using hand nets and traps, and some were obtained from local fishermen.

### DNA Extraction, *COI* Amplification and Sequencing

**FIGURE 1:**
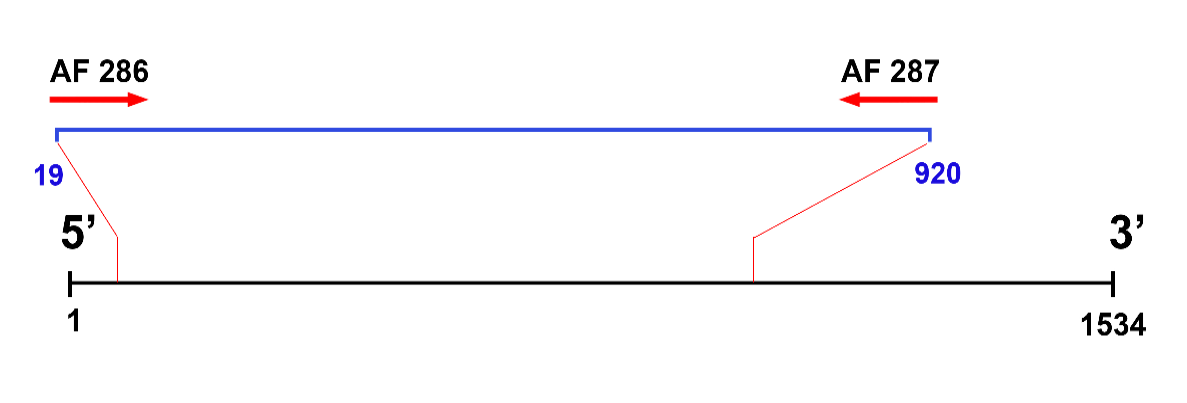
Primer binding site based on the *COI* gene of *Matuta victor* (NC_053638.1).

Genomic DNA was extracted from the muscle tissue of pereiopods using a DNA GeneAid Genomic DNA Mini Kit (manufacturer name, country) following the manufacturer’s protocol. A pair of primers, forward AF286 5′-TCTACAAAYCATAAAGAYATYGG-3′ and reverse AF287 3′-GTGGCRGANGTRAARTARGCTCG-5′ (Rismawati *et al*. 2024), was used for *COI* gene amplification (Fig. 1). Polymerase chain reaction (PCR) was run on BIOMETRA Thermo Cycle with an annealing temperature of 50°C. Tight and single bands of amplification were purified and sequenced by 1st BASE DNA Sequencing Services, Malaysia, using the same primer as in the previous amplification.

### Data Analysis

Sequences were edited in MEGA 11 (Tamura *et al*. 2021) and aligned using the Muscle algorithm with the default option. Population parameters of diversity within each locality are represented as the number of haplotypes (Hn); haplotype diversity (Hd) and nucleotide diversity (π) were estimated using DnaSP 6.0 (Rozas *et al*. 2017). The haplotype networks were constructed using Network 10.2.0.0 (https://www.fluxus-engineering.com). The pairwise fixation index (FST) and analysis of molecular variance (AMOVA) were evaluated in Arlequin 3.5.2.2 (Excoffier & Lischer 2010). Significance of pairwise FST values was tested with 50,000 permutations. To account for multiple pairwise comparisons (m = 45), we applied Holm–Bonferroni correction (adjusted α = 0.05) to the probability values. FST values were calculated based on population differentiation, and statistical tests were used to assess population equilibrium (neutrality test) according to Tajima’s D (1989) and Fu’s Fs (1997).

## RESULTS

Sequence data generated in this study have been deposited in the DNA Data Bank of Japan (DDBJ) with accession numbers LC891144–LC891234.

### Haplotype and Genetic Diversity

A total of 801 bp sequence data was obtained using the pair of primers AF286 and AF287. Of the 91 sequences obtained, 38 haplotypes with 60 polymorphic sites were found on Java Island (see Table S1 in Supplementary Material). Among the polymorphic sites, 42 were parsimonious with two variants, two were parsimonious with three variants, and 18 were singleton with two variants.

**FIGURE 2:**
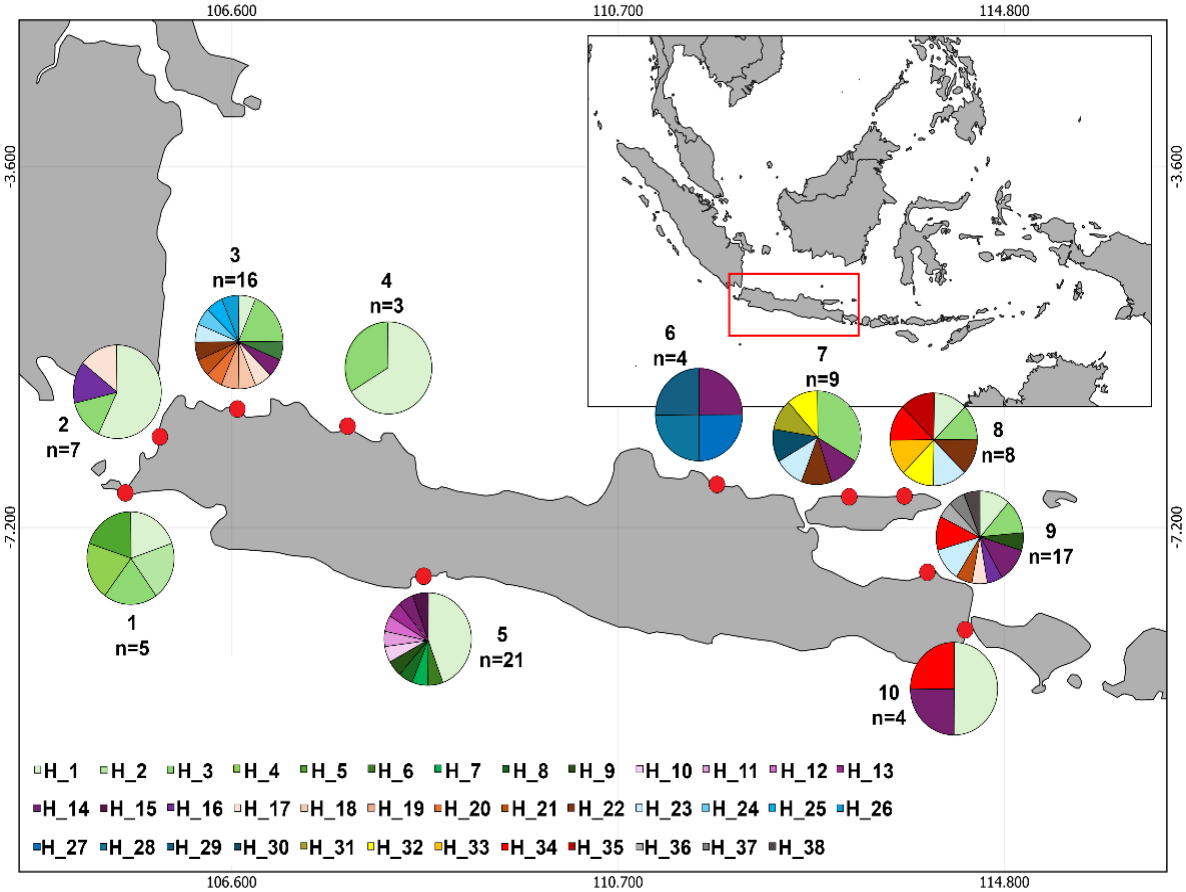
Haplotype of *Matuta victor* from 10 localities on Java Island. 1 = Ujung Kulon; 2 = Carita; 3 = Tanjung Pasir; 4 = Subang; 5 = Pangandaran; 6 = Bancar; 7 = Banyuates; 8 = Ambunten; 9 = Situbondo; 10 = Banyuwangi.

**FIGURE 3:**
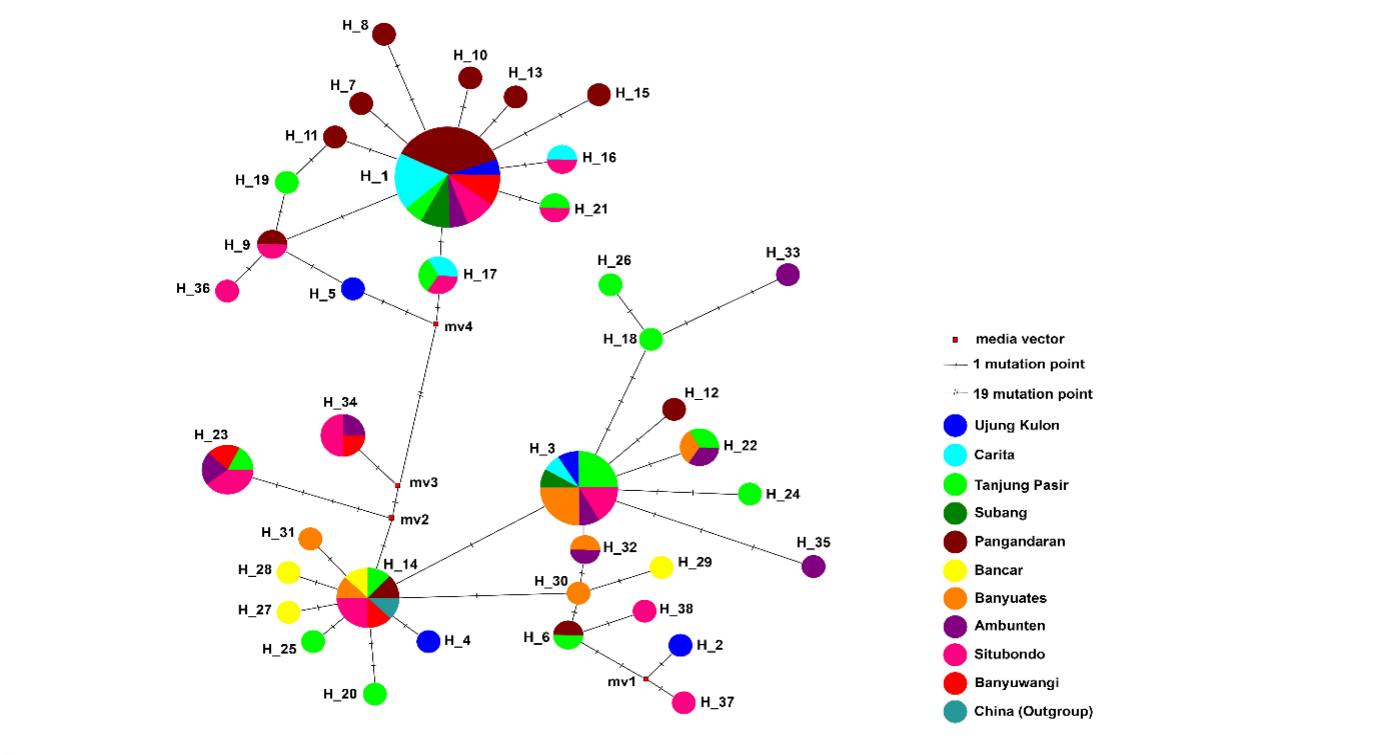
The Median-joining network of *Matuta victor* from 10 localities on Java Island. The smallest circles indicate only one haplotype.

Among the 38 haplotypes, 12 were shared and 26 were unique (Fig. 2). The median-joining network of *M. victor* showed shared and unique haplotypes from 10 localities (Fig. 3). H_1 was the most frequent haplotype, representing 23.07% of the total individuals, and was present in eight localities, namely, Ujung Kulon, Pangandaran, Carita, Tanjung Pasir, Subang, Ambunten, Situbondo and Banyuwangi. Two other locations (Banyuates and Bancar) were shared in H_14. This haplotype is also shared with *M. victor* from China as a reference (NC_053638). All individuals from Carita shared the haplotype with other locations, not just Subang and Banyuwangi. In the haplotype network, 19 mutational steps were detected between haplotypes mv3 and mv4, representing the largest number of mutations observed among all haplotype comparisons.

The haplotype diversity (Hd) of *M. victor* ranged from 0.667–1, with the lowest being from Subang and the highest from Ujung Kulon, Bancar and Ambunten. Nucleotide diversity (π) ranged from 0.003–0.022, with the lowest being from Bancar and the highest from Ujung Kulon, Subang and Banyuwangi (Table 1).

### Genetic Differentiation and Phylogeny

The pairwise FST of *M. victor* between localities ranged from −0.323 to 0.773 (Table 2). The lowest value was between Subang and Banyuwangi, and the highest was between Carita and Banyuates. Based on the AMOVA, 73.39% of the total genetic variation was contributed by within-population variation, and 26.61% was contributed by among-population variations (FST= 0.266) (Table 3). The result pointed to a limited genetic structure of *M. victor* on Java Island, with only a low level of genetic differentiation among 10 localities. Applying Holm–Bonferroni correction for multiple comparisons, five population pairs remained significantly differentiated (Location 1–10, 2–8, 2–10, 3–6, and 4–6). These results indicate that although the overall genetic differentiation among populations of *M. victor* in Java Island is low, certain localities exhibit detectable genetic structure.

The neutrality test showed that Tajima’s D values ranged from −1.518 (*p* < 0.05) to 1.826. Fu’s Fs values ranged from −2.871 to 5.242 (Table 1). While most localities showed non-significant results, significant negative values were detected in Carita (Tajima’s D), as well as Bancar and Banyuates (Fu’s Fs), suggesting signals of population expansion in these areas.

## DISCUSSION

Haplotype diversity is an important measure in population genetics because it reflects the genetic diversity present within a population. Haplotype diversity represents the probability that two randomly sampled alleles are different (Nei 1987). *Matuta victor* from all localities exhibited moderate to high levels of haplotype diversity, while nucleotide diversity was low. The haplotype and nucleotide diversities of this study were like those observed in another brachyuran crab (Ma *et al*. 2011; Sharif *et al*. 2016). The high level of haplotype diversity indicates extensive genetic diversity, resulting in a varied genetic structure (Zamroni *et al*. 2016). Variations in genetic diversity occur when mutations change the order of nucleotide bases in DNA (Handayani *et al*. 2014). The large number of haplotypes suggests that the populations were sizable enough to sustain a significant level of genetic diversity (Aini *et al*. 2021).

Nucleotide diversity, particularly that related to mitochondrial *COI* sequences, provides important insights into the genetic variation within a population. Nucleotide diversity provides a precise parameter of genetic variation, and it remains unaffected by changes in sample size or the length of DNA (Handayani *et al*. 2014). Although nucleotide diversity tends to be low, this does not diminish its importance in depicting overall genetic diversity. Taken together, these results show a consistent pattern of high haplotype diversity but low nucleotide diversity in *M. victor* populations, suggesting substantial genetic variation with relatively limited sequence divergence. This condition may reflect recent population expansion or high gene flow among localities. From a conservation perspective, this highlights the importance of protecting multiple localities, particularly those harbouring unique haplotypes, preserving coastal habitat connectivity to support gene flow.

The AMOVA analysis further supports this interpretation, indicating low genetic differentiation among populations across Java Island. The overall FST value of 0.266 suggests considerable genetic similarity, which may result from extensive genetic exchange between populations. Such genetic homogeneity could be attributed to adult and juvenile migration, high larval dispersal capabilities, and the absence of strong physical barriers in the marine environment (Ma *et al*. 2011).

The sharing of H_1, H_3 and H_14, the three largest haplotypes, among localities may be due to gene flow. Gene flow is the genetic realisation of ongoing patterns of dispersal between populations (Hellberg 2009) and high rates of gene flow are common in marine organisms with large geographic distribution (Crandall *et al*. 2019). Interestingly, the *M. victor* reference from GenBank shared a haplotype with *M. victor* in localities within Java Island. The reference sequence (NC_053638) originated from Beibu Bay, China (Huang *et al*. 2021) suggesting that gene flow across a broad geographic area may shape the distribution pattern of *M. victor*. Brachyuran crabs have a larval phase during their life cycle, and larval dispersal strongly affects biogeographic distribution, population connectivity, genetic diversity and the formation of metapopulations (Anger 2006). The dispersal direction of larvae can be influenced by sea currents (Sanvicente-Anorve *et al*. 2018), and the South China Sea is connected to the Java Sea through the Karimata Strait (Kok *et al*. 2021), potentially facilitating gene flow between *M. victor* populations in Java and China. Consequently, the genetic distribution patterns of *M. victor* can be better understood by studying both gene flow and larval movement in the region (Lu *et al*. 2022).

In addition to haplotype sharing, the haplotype network also revealed 19 mutational steps between mv3 and mv4, representing the highest divergence observed among haplotypes. This relatively large number of mutations may indicate insufficient sampling, where intermediate haplotypes remain undetected, or the possible loss of putative haplotypes in the wild due to demographic fluctuations or local extinctions. Such divergence could also reflect historical separation events followed by secondary contact, or the persistence of rare lineages in certain populations.

All sequences from Carita share haplotypes with other locations, possibly because Carita is located on Sunda Strait, which is the link between the Indian Ocean and the Java Sea. The Sunda Strait is an area with a high level of diversity and abundance (Suharti 1996; Ke *et al*. 2014). The genetic diversity of the blue swimming crab *Portunus pelagicus* indicates no genetic speciation in Sunda Strait (Hidayani *et al*. 2020). These results indicate that gene flow in this location tends to be high, which may be due to the high level of interaction between populations in the Sunda Strait region.

The negative values of Tajima’s D and Fu’s Fs indicate that the tested populations are experiencing population expansion and population growth, leading to an excess of allele numbers due to population expansion (Aris-Brosou & Excoffier 1996). The neutrality test results further support the demographic patterns of *M. victor* in Java. Most populations, including Ujung Kulon, Subang, Situbondo, Banyuwangi, Tanjung Pasir, Pangandaran and Ambunten, exhibited non-significant Tajima’s D and Fu’s Fs values, indicating neutrality or demographic stability, although the negative values observed in some of these localities may reflect potential population expansion. In contrast, Carita showed a significantly negative Tajima’s D value, and Bancar and Banyuates exhibited significantly negative Fu’s Fs values, providing clear signals of population expansion in these areas. These findings are consistent with patterns expected under recent population growth, where excess low-frequency polymorphisms are generated by demographic expansion.

## CONCLUSIONS

Haplotype diversity for each location ranged from moderate to high, while nucleotide diversity was low. The AMOVA analysis showed that the genetic structure of *M. victor* in Java Island is relatively limited, with low genetic differentiation (FST = 0.266) among the 10 studied locations. Neutrality tests further indicated potential population expansion in several localities. Overall, these results reveal that *M. victor* populations in Java exhibit high haplotype diversity but low nucleotide diversity, reflecting the presence of numerous haplotypes with relatively low sequence divergence. Combined with the AMOVA and neutrality test results, this pattern suggests that the populations are genetically homogeneous due to substantial genetic exchange, while some areas may still experience expansion events. These findings provide important insights for conservation, emphasising the need to protect habitats across different localities, preserve unique haplotypes and maintain connectivity between populations to safeguard the long-term genetic resilience of *M. victor* in Indonesia’s coastal ecosystems.

## SUPPLEMENTARY MATERIAL

**TABLE S1:** Polymorphic sites among 38 haplotypes of *M. victor* on Java Island.

Haplotype	N	Nucleotide position (1–30)																													

		4	8	1	1	1	1	1	1	1	2	2	2	2	2	2	2	2	3	3	3	3	3	3	3	3	4	4	4	4	4
		1	3	4	4	5	6	7	7	8	1	1	3	4	5	6	8	9	0	1	4	5	6	7	8	8	1	1	1	3	5
				3	7	5	7	0	3	5	2	5	6	2	7	3	1	6	5	4	1	9	2	7	0	9	3	6	9	4	8
H_1	21	A	C	A	C	A	T	G	T	G	T	A	A	A	T	T	C	T	T	G	G	G	A	A	A	A	G	C	T	G	T
H_2	1	–	–	T	–	G	–	A	–	A	–	G	–	–	–	C	T	C	–	–	A	A	T	–	–	–	A	T	A	A	–
H_3	12	–	–	–	–	–	–	A	–	A	–	G	–	–	–	C	T	C	–	–	A	A	G	–	–	–	A	T	–	A	–
H_4	1	–	–	–	T	–	–	A	–	A	–	G	–	–	–	C	T	C	–	–	A	A	–	–	–	–	A	T	–	A	–
H_5	1	–	–	–	–	–	–	–	–	–	–	–	–	–	–	–	–	–	–	–	–	–	–	–	–	–	A	–	–	–	–
H_6	2	–	–	–	–	G	–	A	–	A	–	G	–	–	–	C	T	C	–	–	A	A	–	–	–	–	A	T	–	A	–
H_7	1	–	–	–	–	–	–	–	–	–	–	–	–	–	–	–	–	–	–	–	–	–	–	–	–	–	–	–	–	–	–
H_8	1	–	–	–	–	–	–	–	–	–	–	–	–	–	–	–	–	–	–	–	–	–	–	–	–	–	–	–	–	–	–
H_9	2	–	–	–	–	–	–	–	–	–	–	–	–	–	–	–	–	–	–	–	–	–	–	–	–	–	A	–	–	–	–
H_10	1	–	–	–	–	–	–	–	–	–	–	–	–	–	–	–	–	–	–	–	–	A	–	–	–	–	–	–	–	–	–
H_11	1	–	–	–	–	–	–	–	–	–	C	–	–	–	–	–	–	–	–	–	–	–	–	–	–	–	–	–	–	–	–
H_12	1	–	–	–	–	–	–	A	–	A	–	G	–	–	–	C	–	C	–	–	A	A	G	–	–	–	A	T	–	A	–
H_13	1	–	–	–	–	–	–	A	–	–	–	–	–	–	–	–	–	–	–	–	–	–	–	–	–	–	–	–	–	–	–
H_14	7	–	–	–	–	–	–	A	–	A	–	G	–	–	–	C	T	C	–	–	A	A	–	–	–	–	A	T	–	A	–
H_15	1	–	–	–	–	–	–	–	–	–	–	–	–	–	–	–	–	–	–	–	–	–	–	–	–	–	–	–	–	–	–
H_16	2	–	–	–	–	–	–	–	–	–	–	–	–	–	–	–	–	–	–	–	–	–	–	–	–	–	–	–	–	–	–
H_17	3	–	–	–	–	–	–	–	–	–	–	–	–	–	–	C	–	–	–	–	–	–	–	–	–	–	–	–	–	–	–
H_18	1	–	–	–	–	–	–	A	–	A	–	–	–	–	G	C	T	C	–	–	A	A	G	–	–	–	A	T	–	A	–
H_19	1	–	–	–	–	–	–	–	–	–	C	–	–	–	–	–	–	–	–	–	–	–	–	–	–	–	A	–	–	–	–
H_20	1	–	–	–	–	–	C	A	–	A	–	G	–	–	–	C	T	C	–	–	A	A	–	G	–	–	A	T	–	A	–
H_21	2	–	–	–	–	–	–	–	–	–	–	–	–	G	–	–	–	–	–	–	–	–	–	–	–	–	–	–	–	–	–
H_22	3	–	–	–	–	–	–	A	–	A	–	G	–	–	–	C	T	C	C	–	A	A	G	–	–	–	A	T	–	A	–
H_23	5	–	–	–	–	–	–	A	–	A	–	G	G	–	–	C	T	–	–	–	A	A	–	–	–	G	A	T	–	A	C
H_24	1	–	–	–	–	–	–	A	–	A	–	G	–	–	–	C	T	C	–	–	A	A	G	–	–	–	A	T	–	A	–
H_25	1	–	–	–	–	–	–	A	–	A	–	G	–	–	–	C	T	C	–	.	A	A	–	–	–	–	A	T	–	A	–
H_26	1	–	–	–	–	–	–	A	–	A	–	–	–	–	G	C	T	C	–	–	A	A	G	–	–	–	A	T	–	A	–
H_27	1	–	–	–	–	–	–	A	–	A	–	G	–	–	–	C	T	C	–	–	A	A	–	–	G	–	A	T	–	A	–
H_28	1	–	T	–	–	–	–	A	–	A	–	G	–	–	–	C	T	C	–	–	A	A	–	–	–	–	A	T	–	A	–
H_29	1	–	–	–	–	–	–	A	–	A	–	G	–	–	–	C	T	C	C	–	A	A	–	–	–	–	A	T	–	A	–
H_30	1	–	–	–	–	–	–	A	–	A	–	G	–	–	–	C	T	C	–	–	A	A	–	–	–	–	A	T	–	A	–
H_31	1	–	–	–	–	–	–	A	–	A	–	G	–	–	–	C	T	C	–	–	A	A	–	–	–	–	A	T	–	A	–
H_32	2	–	–	–	–	–	–	A	–	A	–	G	–	–	–	C	T	C	–	–	A	A	G	–	–	–	A	T	–	A	–
H_33	1	G	–	–	T	–	–	A	–	A	–	–	–	–	G	C	T	C	–	–	A	A	G	–	–	–	A	T	–	A	–
H_34	4	–	–	–	–	–	–	A	–	A	–	G	–	–	–	C	T	–	–	–	A	A	–	–	–	–	A	T	–	A	–
H_35	1	–	–	–	–	–	–	A	–	A	–	G	–	–	–	C	T	C	–	A	A	A	G	–	–	–	A	T	–	A	–
H_36	1	–	–	–	–	–	–	–	–	–	–	–	–	–	–	–	–	–	–	–	–	–	–	–	–	–	A	–	–	–	–
H_37	1	–	–	T	–	G	–	A	A	A	–	G	–	–	–	C	T	C	–	–	A	A	–	–	–	–	A	T	A	A	–
H_38	1	–	–	–	–	G	–	A	–	A	–	G	–	–	–	C	T	C	–	–	A	A	–	–	–	–	A	T	–	A	–

Haplotype	N	Nucleotide position (31–60)																													

		4	4	4	5	5	5	5	5	5	5	5	5	5	5	5	5	5	6	6	6	6	6	6	7	7	7	7	7	7	7
		7	9	9	0	1	1	2	2	3	5	5	6	7	7	9	9	9	0	1	1	5	6	9	0	1	2	4	5	8	9
		9	1	7	0	2	8	2	4	3	4	7	6	5	8	3	6	9	2	1	7	0	8	2	1	9	5	1	5	2	4
H_1	21	T	A	T	C	T	G	C	A	A	T	T	A	C	C	C	G	C	A	C	C	G	T	A	A	C	G	C	T	A	C
H_2	1	C	G	–	T	–	A	T	–	–	–	C	G	–	–	–	–	–	–	T	T	A	–	–	G	T	–	T	–	–	T
H_3	12	C	G	–	T	–	A	T	–	–	–	C	G	T	–	–	–	–	–	T	T	A	–	–	G	T	–	T	–	–	T
H_4	1	C	G	–	T	–	A	T	–	–	–	C	G	T	–	–	–	–	–	T	T	A	–	–	G	T	–	T	–	–	T
H_5	1	–	–	–	–	–	–	–	–	–	–	C	–	–	–	–	–	–	–	–	–	–	–	–	–	–	–	–	–	–	–
H_6	2	C	G	–	T	–	A	T	–	–	–	C	G	–	–	–	–	–	–	T	T	A	–	–	G	T	–	T	–	–	T
H_7	1	–	–	–	–	–	–	–	–	–	–	–	–	–	–	–	–	–	–	T	–	–	–	–	–	–	–	–	–	–	–
H_8	1	–	–	–	–	–	–	–	–	T	–	–	–	–	–	–	–	–	–	–	–	–	–	–	–	–	–	–	–	G	–
H_9	2	–	–	–	–	–	–	–	–	–	–	–	–	–	–	–	–	–	–	–	–	–	–	–	–	–	–	–	–	–	–
H_10	1	–	–	–	–	–	–	–	–	–	–	–	–	–	–	–	–	–	–	–	–	–	–	–	–	–	–	–	–	–	–
H_11	1	–	–	–	–	–	–	–	–	–	–	–	–	–	–	–	–	–	–	–	–	–	–	–	–	–	–	–	–	–	–
H_12	1	C	G	–	T	–	A	T	–	–	–	C	G	T	–	–	–	–	–	T	T	A	–	–	G	T	–	T	–	–	T
H_13	1	–	–	–	–	–	–	–	–	–	–	–	–	–	–	–	–	–	–	–	–	–	–	–	–	–	–	–	–	–	–
H_14	7	C	G	–	T	–	A	T	–	–	–	C	G	T	–	–	–	–	–	T	T	A	–	–	G	T	–	T	–	–	T
H_15	1	–	–	–	–	–	–	–	–	–	–	–	–	–	–	T	–	–	–	–	–	–	–	–	–	–	–	–	C	–	–
H_16	2	–	–	–	–	–	–	–	–	–	–	–	–	–	–	–	A	–	–	–	–	–	–	–	–	–	–	–	–	–	–
H_17	3	–	–	–	–	–	A	–	–	–	–	–	–	–	–	–	–	–	–	–	–	–	–	–	–	–	–	–	–	–	–
H_18	1	C	G	–	T	–	A	T	–	–	–	C	G	T	–	–	–	–	–	T	T	A	C	–	G	T	–	T	–	–	T
H_19	1	–	–	–	–	–	–	–	–	–	–	–	–	–	–	–	–	–	–	–	–	–	–	–	–	–	–	–	–	–	–
H_20	1	C	G	–	T	–	A	T	–	–	–	C	G	T	–	–	–	–	–	T	T	A	–	–	G	T	–	T	–	–	T
H_21	2	–	–	–	–	–	–	–	–	–	–	–	–	–	–	–	–	–	–	–	–	–	–	–	–	–	–	–	–	–	–
H_22	3	C	G	–	T	–	A	T	–	–	–	C	G	T	–	–	–	–	–	T	T	A	–	–	G	T	–	T	–	–	T
H_23	5	C	G	–	–	–	A	T	–	–	–	C	G	T	–	–	–	–	G	T	T	A	–	–	G	T	–	T	–	–	T
H_24	1	C	G	–	T	–	A	T	–	–	–	C	G	T	–	–	–	T	–	T	T	A	–	–	G	T	A	T	–	–	T
H_25	1	C	G	–	T	–	A	T	G	–	–	C	G	T	–	–	–	–	–	T	T	A	–	–	G	T	–	T	–	–	T
H_26	1	C	G	–	T	–	A	T	–	–	–	C	C	T	–	–	–	–	–	T	T	A	C	–	G	T	–	T	–	–	T
H_27	1	C	G	–	T	–	A	T	–	–	–	C	G	T	–	–	–	–	–	T	T	A	–	–	G	T	–	T	–	–	T
H_28	1	C	G	–	T	–	A	T	–	–	–	C	G	T	–	–	–	–	–	T	T	A	–	–	G	T	–	T	–	–	T
H_29	1	C	G	–	T	–	A	T	–	–	–	C	G	–	–	–	–	–	–	T	T	A	–	–	G	T	–	T	–	–	T
H_30	1	C	G	–	T	–	A	T	–	–	–	C	G	–	–	–	–	–	–	T	T	A	–	–	G	T	–	T	–	–	T
H_31	1	C	G	–	T	–	A	T	–	–	–	C	G	T	–	–	–	–	–	T	T	A	–	G	G	T	–	T	–	–	T
H_32	2	C	G	–	T	–	A	T	–	–	–	C	G	–	–	–	–	–	–	T	T	A	–	–	G	T	–	T	–	–	T
H_33	1	C	G	–	T	C	A	T	–	–	–	C	G	T	–	–	–	–	–	T	T	A	C	–	G	T	–	T	–	–	T
H_34	4	C	G	–	–	–	A	T	–	–	C	C	G	T	–	–	–	–	–	T	T	–	–	–	G	T	–	T	–	–	T
H_35	1	C	G	C	T	–	A	T	–	–	–	C	–	T	–	–	–	–	–	T	T	A	–	–	G	T	–	T	–	–	T
H_36	1	–	–	–	–	–	–	–	–	–	–	–	–	–	–	–	–	–	–	–	–	–	C	–	–	–	–	–	–	–	–
H_37	1	C	G	–	T	–	A	T	–	–	–	C	G	–	–	–	–	–	–	T	T	A	–	–	G	T	–	T	–	–	T
H_38	1	C	G	–	T	–	A	T	–	–	–	C	G	–	T	–	–	–	–	T	T	A	–	–	G	T	–	T	–	–	T

*Notes*: Haplotypes are compared with consensus haplotype H_1, “.” indicates identical nucleotides, “N” indicates number of sequences of each haplotype.

## Figures and Tables

**TABLE 1:** Sample size, haplotype diversity and neutrality test of *Matuta victor* on Java Island.

Code	Localities	*n*	Hn	Hd	π	Tajima’s *D*	Fu’s F*S*
1	Ujung Kulon, Pandeglang, Banten	5	5	1	0.022	1.317	0.363
2	Carita, Pandeglang, Banten	7	4	0.714	0.010	−0.443	−0.383
3	Tanjung Pasir, Tangerang, Banten	16	14	0.975	0.016	−1.518*	2.964
4	Subang, West Java	3	2	0.667	0.022	0.166	−2.871
5	Pangandaran, West Java	18	11	0.817	0.011	0	5.242
6	Bancar, Tuban, East Java	4	4	1	0.003	−0.780	−1.872*
7	Banyuates, Sampang, East Java	9	7	0.917	0.003	−1.211	−2.828*
8	Ambunten, Sumenep, East Java	8	8	1	0.014	−1.337	−1.877
9	Situbondo, East Java	17	12	0.963	0.019	1.313	0.271
10	Banyuwangi, East Java	4	3	0.833	0.022	1.826	3.574
	All localities	91	38	0.921	0.018		

**TABLE 2:** Pairwise FST of *Matuta victor* from 10 localities on Java Island.

Locality	1	2	3	4	5	6	7	8	9	10
1										
2	0.235									
3	−0.076	0.406								
4	−0.161	−0.179	0.116							
5	0.280	−0.094	0.433	−0.124						
6	0.149	0.734	0.027*	0.521*	0.687					
7	0.274	0.773	0.079	0.631	0.718	0.032				
8	−0.016	0.530*	−0.058	0.237	0.546	−0.015	−0.004			
9	−0.113	0.199	0.014	−0.093	0.237	0.182	0.252	0.063		
10	−0.193*	0.056*	0.019	−0.323	0.100	0.345	0.480	0.109	−0.146	

*Notes*:

* Significant after Holm–Bonferroni correction for multiple comparisons (m = 45, adjusted α = 0.05). 1 = Ujung Kulon; 2 = Carita; 3 = Tanjung Pasir; 4 = Subang; 5 = Pangandaran; 6 = Bancar; 7 = Banyuates; 8 = Ambunten; 9 = Situbondo; 10 = Banyuwangi.

**TABLE 3:** Analysis of molecular variance (AMOVA) of mtDNA *COI* sequence of *Matuta victor* on Java Island.

Source of variation	d.f.	Percentage of variation	FST	*p*-value
Among populations	9	26.61	0.266	0.000
Within population	81	73.39		
Total	90			
